# Scrub Typhus in Continental Chile, 2016–2018^1^

**DOI:** 10.3201/eid2506.181860

**Published:** 2019-06

**Authors:** Thomas Weitzel, Constanza Martínez-Valdebenito, Gerardo Acosta-Jamett, Ju Jiang, Allen L. Richards, Katia Abarca

**Affiliations:** Clinica Alemana, Universidad del Desarrollo, Santiago, Chile (T. Weitzel);; Pontificia Universidad Católica de Chile, Santiago (C. Martínez-Valdebenito, K. Abarca);; Universidad Austral de Chile, Valdivia, Chile (G. Acosta-Jamett);; Naval Medical Research Center, Silver Spring, Maryland, USA (J. Jiang, A.L. Richards)

**Keywords:** scrub typhus, *Orientia* species, epidemiology, South America, Chile, vector-borne infections, zoonoses, rickettsia, bacteria

## Abstract

Endemic scrub typhus was recently detected on Chiloé Island in southern Chile. We report a series of cases, acquired over a wide geographical range in continental Chile during 2016–2018, demonstrating that this emerging rickettsial infection is also found on the mainland of South America.

Scrub typhus is a vectorborne zoonosis caused by *Orientia* spp. bacteria; infection carries a potentially severe outcome (*1*). Although widely underrecognized, scrub typhus is considered one of the most important rickettsial infections worldwide in terms of prevalence and severity (*2*). Until recently, scrub typhus was associated with only a single species, *O. tsutsugamushi*, which is transmitted by larvae of trombiculid mites (chiggers) and threatens >1 billion human inhabitants within the so-called tsutsugamushi triangle in the Asia–Pacific region (*1*). Since 2006, the discoveries of scrub typhus in 4 patients on Chiloé Island in Chile (*3**,**4*) and in 1 patient from Dubai, United Arab Emirates (*5*), have suggested the emergence of the disease farther afield (*6*). This change of paradigm has been reinforced by recent studies mainly from Africa (*7*). The emergence of endemic scrub typhus on Chiloé Island has been confirmed by ongoing studies of our working group in Chile (*8**,**9*). Whether this disease is only endemic to Chiloé Island or has a wider distribution is unknown. We report 9 patients who had scrub typhus diagnosed after visiting different regions of continental Chile during 2016–2018.

## The Study

After the confirmation of autochthonous scrub typhus cases in Chiloé in 2016 (*4*), the Chilean Ministry of Health issued a clinical alert advising healthcare providers in Chile to confirm possible cases in cooperation with our research group. Most of the patients described in this report were identified as a result of the clinical alert and are included in an ongoing clinical–epidemiologic scrub typhus project. 

We tested serum samples obtained during acute and convalescent phases of infection for *Orientia*-specific antibodies by using a commercial IgG indirect immunofluorescence assay (Fuller Laboratories, http://www.fullerlabs.com), based on whole-cell *O. tsutsugamushi* Gilliam, Karp, Kato, and Boryong strains, and by using Scrub Typhus Detect IgG and IgM ELISA (InBios International Inc., http://www.inbios.com) with recombinant 56-kD type–specific antigens of *O. tsutsugamushi* Karp, Kato, Gilliam, and TA716 strains. We examined DNA extracted from eschar material by using 16S rRNA (*rrs*) and 47 kDa gene (*htrA*) seminested PCR and sequencing, as previously described (*10*), except that we changed the forward primer of the PCR step of *rrs* from 16SU17 to 16SOR155f. We also tested all samples by using a recently developed *Orientia* genus–specific quantitative real-time PCR assay targeting *rrs* (*11*).

Among the 37 patients with suspected scrub typhus who were tested during 2016–2018, 13 were from mainland Chile. Of those, 9 had scrub typhus diagnosed both serologically and molecularly. None of these 9 patients lived in or traveled to Chiloé Island or other regions with endemic scrub typhus, but all were exposed to natural habitats on mainland Chile (Table 1). Seven patients were male and 2 female; median age was 28 years. 

**Table 1:** Demographic and epidemiologic data of 9 scrub typhus cases in continental Chile, 2016–2018

Case no.	Age, y/sex	Month of probable exposure	Location of probable exposure	
			Region	Site
1	43/M	2016 Mar	Aysén	Caleta Tortel
2	56/M	2017 Feb	Los Lagos	Pumalín
3	25/M	2017 Mar	Los Lagos	Cochamó
4	69/M	2018 Feb	Aysén	Queulat
5	22/F	2018 Feb	Los Lagos	Puelo
6	25/M	2018 Feb	Los Lagos	Puelo
7	39/M	2018 Feb	Los Lagos	Tagua Tagua
8	28/M	2018 Mar	Bío Bío	Alto Bío Bío
9	21/F	2018 Mar	Los Lagos	Cochamó

All cases occurred after outdoor activities during the summer months of February and March. We defined probable exposure sites as those locations where patients reported outdoor activities with close contact to natural environments within the 7–20 days before symptom onset. Most infections were acquired in the Los Lagos Region, which includes Chiloé Island. However, 2 cases were acquired farther south, in the Aysén Region, and another case farther north, in the Bio Bío Region (Figure). The sites of exposure ranged over a total distance of >1,120 km (https://ec.europa.eu/programmes/erasmus-plus/resources/distance-calculator_en), from 38°03′S to 47°47′S. Details of the activities leading to the exposure have been described elsewhere (*12*). 

**Figure:**
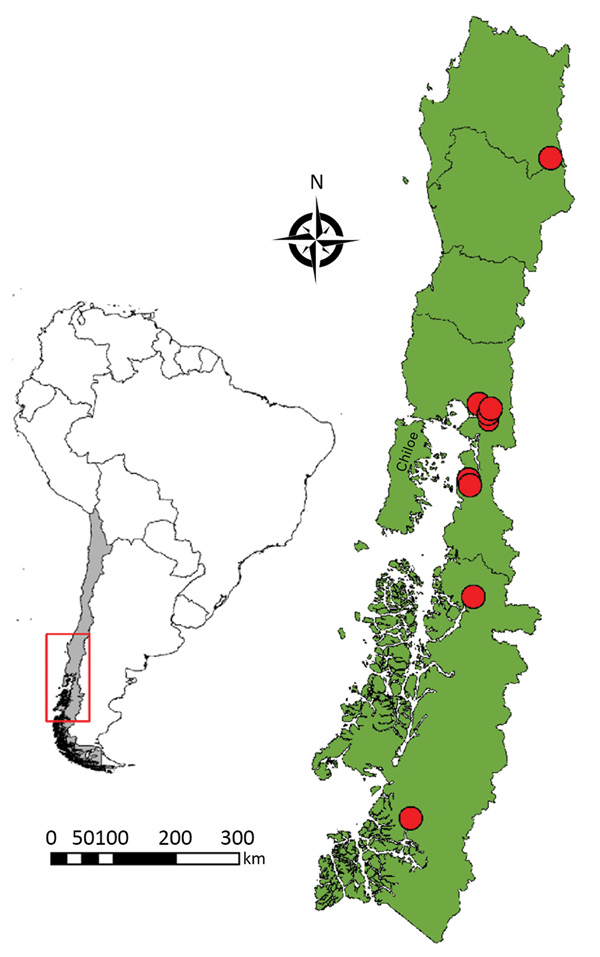
Locations of probable exposures (red circles) of 9 scrub typhus patients in continental Chile, 2016–2018. Inset map shows the study area (red box) and the location of Chile (gray shading) within South America.

All patients had fever, generalized maculopapular rash, eschar, and headache; other frequent symptoms were myalgia (8 patients) and regional lymphadenopathy (5 patients). Laboratory abnormalities included elevated C-reactive protein (8 patients) and transaminases (6 patients), thrombocytopenia (4 patients), and leukopenia (4 patients). Eight patients required hospitalization. We noted a serologic response to *O. tsutsugamushi* antigens in all 9 patients. Eight patients were positive for IgG by indirect immunofluorescence assay, mostly with low titers, and 3 of the 6 patients for whom convalescent-phase samples were available showed seroconversion or a >4-fold rise in titer. IgG and IgM results by ELISA were positive in 6 of 9 patients on the basis of local cutoff values (Table 2). All cases were confirmed by detection of *Orientia*-specific DNA from eschar material using 3 different PCR assays (Table 2). After treatment with doxycycline (7 patients) or azithromycin (1 patient), patients recovered rapidly; 1 patient recuperated after 6 days of fever without receiving any scrub typhus–specific treatment.

**Table 2:** Serologic and molecular diagnosis of scrub typhus cases in continental Chile, 2016–2018*

Case no.	Serologic testing results											
	IFA IgG†			ELISA IgG‡			ELISA IgM‡			Eschar molecular testing results		
	Acute	Conv		Acute	Conv		Acute	Conv		PCR *rrs*	PCR 47kDa	qPCR *rrs*
1	256	256		+	+		+	–		+	+	+
2	<32	128		–	–		–	–		+	+	+
3	64	512		–	+		+	–		+	+	+
4	128	256		–	–		–	–		+	+	+
5	128	NA		+	NA		+	NA		+	+	+
6	128	NA		–	NA		–	NA		+	+	+
7	128	256		–	+		+	+		+	+	+
8	<32	NA		+	NA		+	NA		+	+	+
9	64	256		–	+		–	+		+	+	+

*Acute, acute-phase; conv, convalescent-phase; NA, not available; qPCR, quantitative PCR; +, positive; –, negative. †Highest titer among 4 antigens. ‡Cutoff values calculated as mean optical density of negative local population + 3 SD.

## Conclusions

Our understanding of the global epidemiology of *Orientia* spp. as human pathogens has undergone important changes. Most importantly, the paradigm of the geographic limitation of scrub typhus to the Asia–Pacific region, which had been unchallenged since the first scientific description of this disease at the beginning of the last century, has been superseded in light of the recent identification of scrub typhus cases on the Arabian Peninsula (Dubai) and South America (Chiloé Island in Chile) (*4**,**5*). The Dubai patient was infected by a distinct *Orientia* species, *Candidatus* O. chuto; the complete description of the isolates from Chile is pending. 

Further studies in rodents and vectors have demonstrated molecular evidence of *Orientia* spp. or *Orientia*-like organisms in South Africa, Kenya, Senegal, and France (*6**,**13**,**14*). In addition, serologic reports point to possible human exposure to *Orientia* microorganisms in Djibouti, Kenya, Republic of the Congo, Cameroon, and Peru (*6**,**7**,**15*). Current data suggest that *Candidatus* O. chuto might be the *Orientia* species from Africa and the Arabian Peninsula (*5**,**14*). Clinical data from these regions are scarce, but 1 molecularly proven case and 2 cases diagnosed on the basis of serologic results occurred in patients with typical symptoms of scrub typhus (*5**,**6*).

The case series we report provides important epidemiologic information for South America, highlighting that scrub typhus is not limited to Chiloé Island but also occurs over a wide range of continental Chile. The actual incidence of the infection remains unknown; the observed increase from 1 case in 2016 to 6 cases in 2018 most probably reflects the growing awareness of scrub typhus among infectious diseases physicians in Chile. Because all of the case-patients we describe were included through a surveillance based on passive case detection, we believe that they only represent the tip of the iceberg for scrub typhus in Chile and South America.

The clinical data of this series showed the classical scrub typhus manifestation: fever, generalized maculopapular rash, and eschar at the inoculation site. However, this finding has to be interpreted cautiously because our epidemiologic alert describes these manifestations as typical, which biases the physicians’ attention toward these presentations. Less typical clinical signs and symptoms (e.g., without rash or with predominant respiratory symptoms), which are present in Asia in a relevant percentage of cases (*1*), might go undiagnosed in Chile. 

All patients were serologically positive according to >1 of the applied commercial assays, which were based on *O. tsutsugamushi* antigens. The low seroreactivity against these antigens, however, suggests a distinct strain or species, which is supported by our preliminary molecular analyses (data not shown). Eschar material, which can be stored and transported under simple conditions (i.e., in a dry plastic tube at 4°C), proved to be the best and most practical specimen, permitting a rapid and reliable confirmation by molecular methods. Studies are ongoing to culture the *Orientia* species in Chile and to understand its life cycle, including vectors and possible zoonotic reservoirs.
